# Atorvastatin improves the proliferation and migration of endothelial progenitor cells via the miR-221/VEGFA axis

**DOI:** 10.1042/BSR20193053

**Published:** 2020-11-25

**Authors:** Lihua Sun, Ying Zhang, Junshi Zhang, Juan Wang, Shifeng Xing

**Affiliations:** 1 Department of Cardiology, Fifth Affiliated Hospital of Xinjiang Medical University, Urumqi 830011, China; 2 Department of Hypertention, First Affiliated Hospital of Xinjiang Medical University, Urumqi 830054, China

**Keywords:** atorvastatin, coronary slow flow (CSF), endothelial progenitor cells (EPCs), miR-221, VEGFA

## Abstract

The present study was aimed at investigating the detailed functions of atorvastatin, a lipid-lowering agent, in the pathogenesis of coronary slow flow (CSF), a clinical disease characterized by delayed angiographic coronary opacity without obstructive coronary disease. In the present study, we successfully identified isolated endothelial progenitor cells (EPCs) from the peripheral blood of patients with CSF. Their vascular endothelial growth factor-A (VEGFA) protein levels were determined using immunoblotting analyses. We determined cell viability using MTT assays, cell migration capacity using Transwell assays, and the angiogenic capacity using a tube formation assay. The target association between miR-221 and VEGFA was validated with a luciferase reporter assay. Atorvastatin treatment increased EPC VEGFA protein levels, proliferation, migration, and angiogenesis. miR-221 expression was down-regulated after atorvastatin treatment; miR-221 overexpression exerted an opposing effect to atorvastatin treatment on VEGFA protein, EPC proliferation, migration, and angiogenesis. The protective effects of atorvastatin treatment on VEGFA protein and EPCs could be significantly suppressed by miR-221 overexpression. miR-221 directly bound the VEGFA 3′UTR to inhibit its expression. In conclusion, atorvastatin improves the cell proliferation, migration, and angiogenesis of EPCs via the miR-221/VEGFA axis. Thus, atorvastatin could be a potent agent against CSF, pending further *in vivo* and clinical investigations.

## Introduction

Coronary slow flow (CSF) is a clinical disease characterized by delayed angiographic coronary opacity without obstructive coronary disease [1,2] and is often associated with chest pain. CSF has been associated with a number of clinical features, including cardiac ischemia, life-threatening cardiac arrhythmias, and heart-related sudden death [3,4]. CSF has also been associated with inflammation, microvascular disease, blood vessel endothelium dysfunction, impaired glucose tolerance, and other diseases [5], but its pathogenesis remains unclear.

The statin atorvastatin is commonly prescribed as a lipid-lowering agent. Atorvastatin has been demonstrated to be useful for dyslipidemia treatments and to prevent cardiovascular disease [6,7]. In the past decades, its role in CSF has been under investigation. Many studies have shown that a long-term administration of atorvastatin could provide beneficial effects to the coronary blood flow and coronary blood reserve in patients with CSF [8,9]. Short-term lipid-lowering therapy (atorvastatin) could promote coronary flow reserve and coronary microvascular function [8]. Nevertheless, the potential mechanisms of atorvastatin’s action on CSF remain largely unknown.

The integrity and functions of the endothelial monolayer cells play a crucial role in the prevention of atherosclerosis [10]. Circulating endothelial progenitor cells (EPCs) are thought to be essential for maintaining the integrity of the endothelium and for replacing apoptotic and impaired endothelial cells, thereby preventing different cardiovascular diseases [11,12]. The reduction in the numbers of EPCs is related to cardiovascular morbidity in patients [13]. Interestingly, 3-hydroxy-3-methylglutaryl-coenzyme A reductase inhibitors (statins) have been shown to increase the number of EPCs of patients with coronary artery disease (CAD) [14]. In addition, as reported in reported by an *ex vivo* study with on EPCs, statins can prevent EPC aging and promote cell growth and colony formation [15]. Based on these findings, we hypothesized that atorvastatin may improve the proliferation and migration of EPCs to exert protective effects on patients with CSF.

Angiogenesis is associated with collateral vessel development in CAD [16]. As a key regulatory factor of physiological angiogenesis [17,18], vascular endothelial growth factor-A (VEGFA) could be associated with CAD [19,20]. Moreover, VEGFA is a growth factor for endothelial cells and a migration factor for smooth muscle cells. In addition to the regulation by protein-coding RNAs, a family of non-coding small RNAs, namely microRNAs (miRNAs), can degrade target mRNAs or inhibit their translation to reduce gene expression [8,9], and participate in both normal physiological activities and pathological processes. According to a large-scale analysis of miRNA expression in human blood vessel endothelium, miR-221 plays a role during angiogenesis [10]. In addition, miR-221 can regulate CD34-positive hematopoietic progenitor cell growth and differentiation [11]. More importantly, online tools predict that miR-221 may exert a negative regulatory effect on the expression of VEGFA via binding to its 3′UTR. Thus, we hypothesized that miR-221 and VEGFA may be associated with atorvastatin through mechanisms affecting the growth and migratory capacity of EPCs.

Herein, we isolated EPCs from peripheral blood of patients with CSF and identified them by immunofluorescence (IF) staining. We determined the effects of atorvastatin on VEGFA protein levels, as well as on the growth and migratory capacity of the EPCs. Next, we evaluated the cellular effects of miR-221 on the same cellular variables in the absence or presence of atorvastatin treatment. Finally, we examined the putative binding of miR-221 to VEGFA. With our experiments, we demonstrated a new mechanism by which atorvastatin may improve the growth and migratory capacity of EPCs by miRNA modulation.

## Materials and methods

### Isolation and identification of EPCs

A total of 20 consecutive patients with CSF were recruited with the approval of the Ethics Committee of The Fifth Affiliated Hospital of Xinjiang Medical University (XYDWFYLS-2019-08). At the same time, 20 contemporary patients with angiographically normal coronary flow were recruited as controls. The exclusion criteria were adopted from those in a previous study [21]. Written informed consents were obtained from all patients enrolled.

We collected fasting 10 ml peripheral blood samples from all the study participants in the morning. The peripheral blood mononuclear cells were isolated by Ficoll gradient centrifugation and then inoculated on to a culture plate coated with human fibronectin (BD, U.S.A.). The M199 medium supplemented with 20% foetal bovine serum (Thermo Fisher Scientific, Waltham, MA, U.S.A.), 30 μg/ml endothelial cell growth supplements (Sigma–Aldrich, St. Louis, MO, U.S.A.), 90 μg/ml heparin (Selleck Chemicals, Houston, TX, U.S.A.), and 1% antibiotics solution was changed every 3 days. The adherent cells were screened for markers of peripheral blood EPCs, 7 days later. We identified Dil-AcLDL and FITC-UEA-I (FITC-lectin) (Sigma–Aldrich) double-stained positive cells as differentiated EPCs by IF staining. The purity of isolated EPCs was determined by flow cytometry using anti-CD34 and anti-VEGFR antibodies.

### Cell treatment and transfection

For atorvastatin treatment, we exposed EPCs to 1 μM of atorvastatin for 24 h. Cells were harvested for further experiments. For cell transfection, we transfected EPCs with the miR-221 mimics, inhibitors, or negative control (NC) RNA using Lipofectamine 2000 (Invitrogen, U.S.A.). After 24 h, we treated the transfected EPCs with or without atorvastatin for another 24 h. Next, we harvested the cells for further experiments.

### Real-time PCR

Total RNA was extracted using TRIzol reagent (Invitrogen) following the manufacturer’s protocol. The miRNA and mRNA real-time PCR analyses were performed following methods described previously [22] using the miScript Reverse Transcription kit (Qiagen, Germany) and the SYBR Green PCR Master Mix (Qiagen). We used U6 and GAPDH expressions as endogenous controls, and applied the 2^−ΔΔ*C*_T_^ method to analyze the relative fold changes in expression.

### Immunoblotting analyses

The cellular protein levels of VEGFA were determined using immunoblotting analyses following methods described previously [22] using anti-VEGFA (ab1316, Abcam, Cambridge, MA, U.S.A.), anti-GAPDH (ab8245, Abcam), and a proper HRP-conjugated secondary antibody. We visualized signals using enhanced chemiluminescence (ECL) substrates (Millipore, U.S.A.). We normalized protein expressions to that of the endogenous GAPDH.

### MTT assay

The cell viability was determined using an MTT assay following methods described before [22]. We seeded cells on to 96-well plates (5 × 10^3^ cells/well) and, 24 h later, transfected and/or treated them as described. Forty-eight hours after transfection, we added 20 μl of MTT (at a concentration of 5 mg/ml; Sigma–Aldrich), and incubated the cells for an additional 4 h in a humidified incubator. The supernatants were discarded and 200 μl of DMSO were added to dissolve the formazan. OD values were measured at 490 nm. We calculated the viability of cells from all other groups, by comparison with the cell numbers of the control group (non-treated cells).

### Migration capacity determined by Transwell assays

We plated cells on the top side of polycarbonate Transwell filters without a Matrigel in the top chamber of the QCM 24-well (Cell Biolabs, San Diego, CA, U.S.A.) at a density of 5 × 10^5^ using medium without serum; we added medium with serum to the bottom chamber. After discarding the non-migratory cells, we fixed the migrated cells on to the lower membrane surface, stained them with Crystal Violet (Beyotime Institute of Biotechnology, Haimen, China), and counted their numbers under a microscope.

### Luciferase reporter assay

To confirm the predicted binding of miR-221 and VEGFA, we subcloned a 3′UTR VEGFA fragment downstream of the *Renilla* gene in the psiCHECK2 vector (Promega, Madison, WI, U.S.A.) and called the new vector wt-VEGFA 3′UTR. The predicted miR-221 binding site was mutated in the mutant reporter vectors and named mut-VEGFA 3′UTR. We co-transfected HEK293 cells (ATCC) and used the Dual Luciferase Reporter Assay System (Promega) to determine their luciferase activity. We normalized the *Renilla* luciferase activity to that of the firefly luciferase for each transfected well.

### Tube formation assay

We assessed the neovascularization capacity of EPCs using an *in vitro* tube formation assay. We plated Matrigels into 96-well plates at 37°C for up to 1 h to form a reconstituted basement membrane. Atorvastatin-treated EPCs were harvested and resuspended (1× 1 0^4^ cells per 100 μl medium), seeded on Matrigel, and incubated at 37°C for 6 h. We inspected the tube structures under an inverted light microscope.

### Chick allantoic membrane assay

We incubated fertilized chicken eggs at 37°C with 70% humidity for 10 days. After localizing the vascularized areas of the chick allantoic membrane (CAM), we drilled a hole above the vascularized areas, penetrating through the outer shell membrane without injuring the CAM. We placed sterilized scraps of filter paper absorbed with 1 μM of atorvastatin or without atorvastatin on top of the CAM in the corresponding groups eggs. Then, we sealed the eggs and incubated them for more 5 days. The vascularized areas of CAM were washed with PBS and photographed. The Ethics Committee of The Fifth Affiliated Hospital of Xinjiang Medical University approved all the experiments (XYDWFYLS-2019-08).

### Statistical analysis

We processed data from at least three independent experiments using SPSS17.0 (IBM, Armonk, NY, U.S.A.) and expressed them as means ± standard deviation (SD). We used a Student’s *t* test to compare the differences between means and a one-way ANOVA to compare the differences among multiple groups. *P<0.05; **P<0.01.

## Results

### Atorvastatin promotes VEGF expression and improves the growth and migratory capacity of EPCs

**Figure 1:**
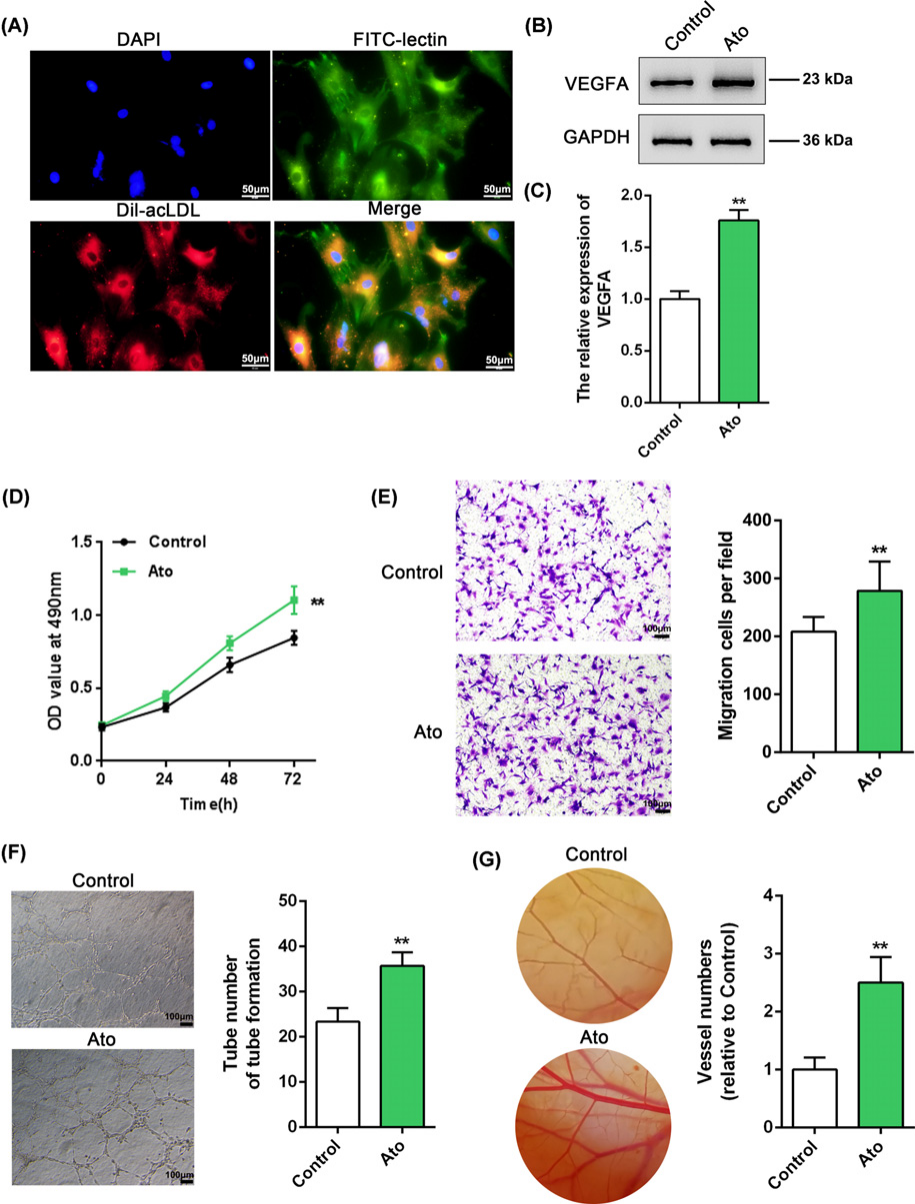
Atorvastatin promotes VEGF expression and improves the proliferation and migration of EPCs (**A**) Isolated EPCs were identified by examining the Dil-acLDL (24 μg/ml) and FITC-UEA-I (FITC-lectin) (10 μg/ml) via IF staining. (**B**–**E**) EPCs were treated with or without atorvastatin (Ato) and we used immunoblotting to quantitate VEGFA protein levels (B,C); (D) MTT cell viability assays; (E) Transwell cell migration assays. (**F**) Tube formation assay applied to detect the effect of atorvastatin on the angiogenic capacity of EPCs *in vitro*. (**G**) CAM assay to investigate the effect of atorvastatin on blood vessel formation of EPCs *in vivo.* **P<0.01.

To investigate the effects of atorvastatin on EPC proliferation and migration, we first isolated EPCs from the peripheral blood of patients with CSF and identified them by IF staining. As shown in Figure 1A, the EPCs showed Dil-AcLDL and FITC-UEA-I (FITC-lectin) double-stained positivity. Next, we treated EPCs with atorvastatin, quantified their VEGFA protein levels, and assessed their growth and migratory capacity. As shown in Figure 1B–E, atorvastatin treatment significantly induced VEGFA protein expression, and enhanced the growth and migratory capacity of the cells. Moreover, we assessed the effects of atorvastatin on the angiogenic capacity of EPCs *in vitro* and found that the tube number was increased in atorvastatin-treated EPCs (Figure 1F). To investigate the effect of atorvastatin on blood vessel formation *in vivo*, we photographed the vascularized areas of CAM. The blood vessel number was markedly increased in CAMs treated with atorvastatin compared with the number in the control group (Figure 1G). These data indicate that atorvastatin treatment could improve growth, and the migratory and angiogenic capacity of EPCs, probably in a VEGFA-related manner.

### miR-221 expression is up-regulated in CSF-associated EPCs and miR-221 inhibits EPC growth and migratory capacity

**Figure 2:**
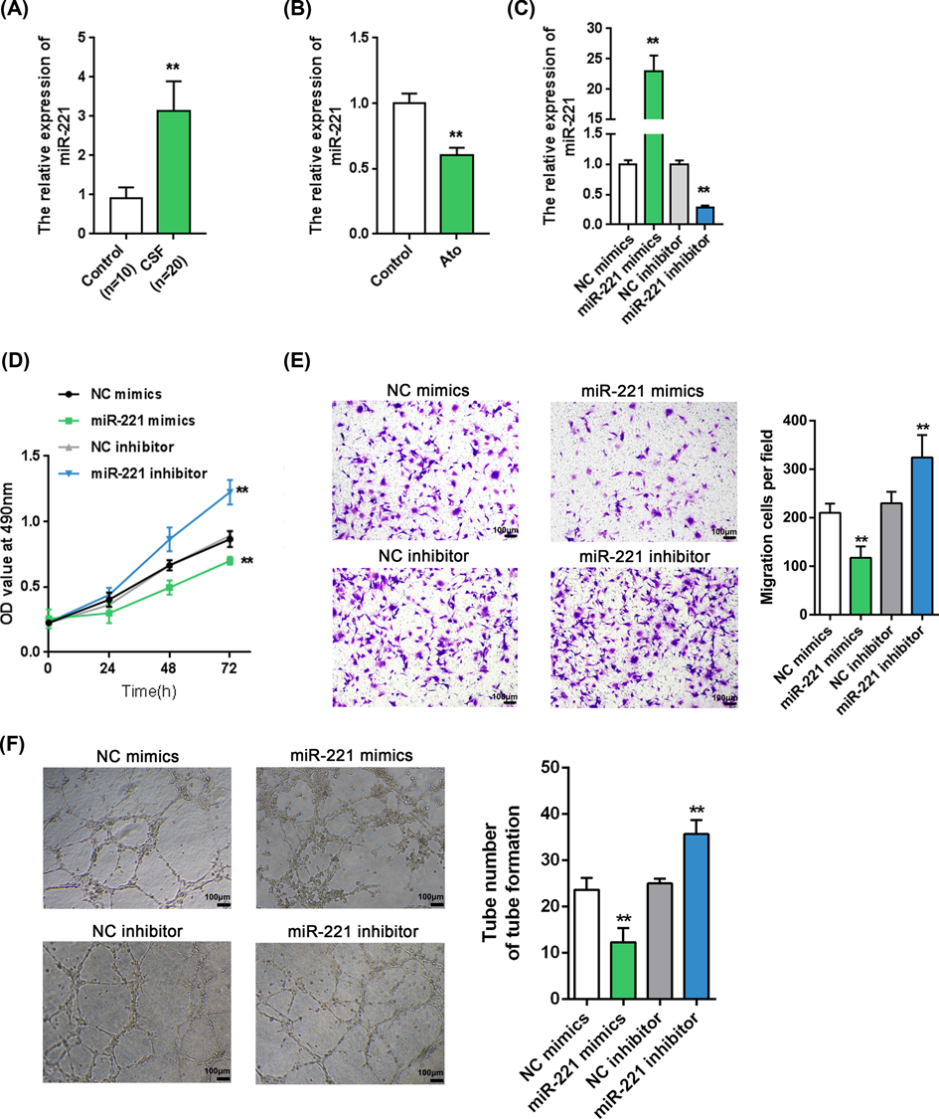
miR-221 expression is up-regulated in CSF and miR-221 inhibits EPC proliferation and migration (**A**) We examined miR-221 expression in EPCs from 10 normal controls and 20 patients with CSF by real-time PCR. (**B**) EPCs were treated with atorvastatin (Ato) and examined for miR-221 expression. (**C**) miR-221 expression in EPCs after transfection of miR-221 mimics or inhibitor, as confirmed by real-time PCR. (**D**) MTT assays to determine cell viability of miR-221-overexpressing or miR-221-silenced EPCs. (**E**) Transwell assays to determine cell migration of miR-221-overexpressing or miR-221-silenced EPCs. (**F**) Tube formation assay to detect the angiogenic capacity of miR-221-overexpressing or miR-221-silenced EPCs. *P<0.05, **P<0.01.

As mentioned, miR-221 affects angiogenesis [23]. The deregulation of miR-221 expression is significantly associated with CAD [24–27]. We investigated miR-221 expression in vivo and in vitro, as well as its cellular functions. Consistent with previous studies, miR-221 expression in isolated EPCs from patients with CSF could be remarkably increased to levels higher than the level in normal control cells (Figure 2A). At the same time, the miR-221 expression in EPCs could be significantly suppressed by atorvastatin treatment (Figure 2B), suggesting that miR-221 may be involved in the atorvastatin improvement of EPC growth and migratory capacity.

Next, we evaluated the cellular effects of miR-221 on EPCs. We transfected miR-221 mimics/inhibitor to conduct miR-221 overexpression/inhibition experiments in EPCs, and performed real-time PCR to verify the transfection efficiency (Figure 2C). We found, by MTT and Transwell assays, that miR-221 overexpression significantly down-regulated the EPC growth and migratory capacity, while miR-221 silencing up-regulated them (Figure 2D,E). In addition, tube formation assay results showed that miR-221 mimics observably restrained the angiogenic capacity of EPCs, while miR-221 knockdown promoted it (Figure 2F).

### Atorvastatin inhibits miR-221 and promotes VEGFA to regulate EPC proliferation and migration

**Figure 3:**
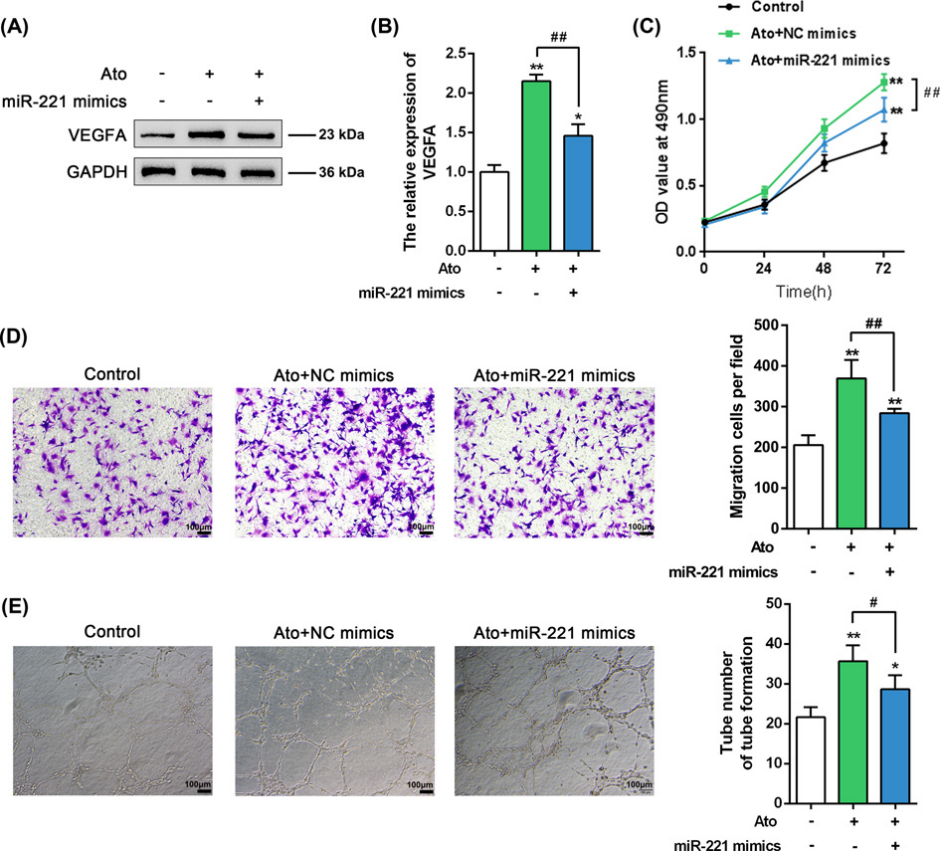
Atorvastatin inhibits miR-221 and promotes VEGFA expression to regulate EPC proliferation and migration (**A,B**) Immunoblotting to quantitate VEGFA protein levels of EPCs transfected with miR-221 mimics with or without atorvastatin (Ato) treatment; (**C**) MTT cell viability assays; (**D**) Transwell cell migration assays; (**E**) Tube formation assay to assess angiogenic capacity. *P<0.05, **P<0.01, compared to control group. #*P*<0.05, ##*P*<0.01, compared to Ato treatment group.

To provide evidence on miR-221’s involvement in the atorvastatin effects on EPCs, we transfected EPCs with miR-221 mimics. Moreover, we quantified VEGFA protein levels, and we assessed cell growth and cell migration in the presence or absence of atorvastatin. The overexpression of miR-221 significantly decreased the atorvastatin-induced increase in the VEGFA protein levels (Figure 3A,B), and it remarkably inhibited the atorvastatin-rescued EPCs cell viability, migration, and angiogenesis (Figure 3C-E). These data indicate that miR-221 could mediateatorvastatin effects on EPCs, most possibly via regulation of VEGFA expression.

### miR-221 negatively regulate the expression of VEGFA by binding to its 3′UTR

**Figure 4:**
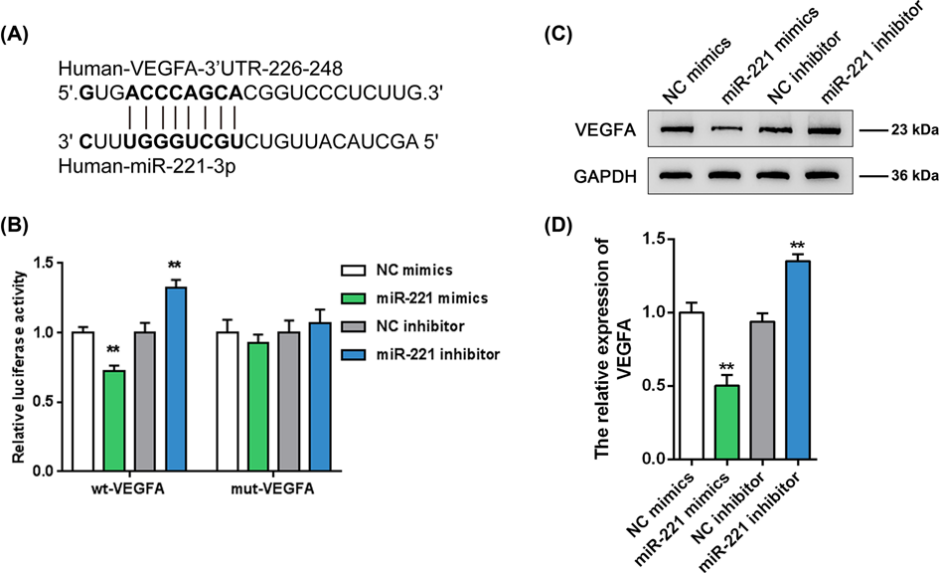
miR-221 targets the VEGFA 3′UTR to negatively regulate its expression (**A**) Schematic diagram showing the predicted binding site between miR-221 and the VEGFA 3′UTR and the structures of wild- and mutant-type VEGFA 3′UTR luciferase reporter vectors. (**B**) HEK293 cells were co-transfected with these vectors and with miR-221 mimics or inhibitor and examined for luciferase activity. (**C,D**) We measured VEGFA protein levels of EPCs transfected with miR-221 mimics or inhibitor. **P<0.01.

We used online tools to predict a putative miR-221 binding site on the VEGFA 3′UTR; therefore, we conducted a luciferase reporter assay by constructing two different types of luciferase reporter vectors with either wild- or mutant-type VEGFA 3′UTRs (namely wt-VEGFA 3′UTR and mut-VEGFA 3′UTR). The putative miR-221 binding site in the mutant-type VEGFA 3′UTR reporter vector was mutated to remove the complementarity with miR-221 (Figure 4A). Afterward, we co-transfected these vectors into HEK293 cells with either miR-221 mimics or inhibitor and examined the luciferase activity of the transfected cells. According to Figure 4B, miR-221 overexpression significantly down-regulated the luciferase activity of the wild-type VEGFA 3′UTR, while miR-221 silencing up-regulated it; moreover, mutating the putative miR-221 binding site could abolish the alterations in the luciferase activity. In summary, miR-221 may directly target the VEGFA 3′UTR. Consistently, miR-221 exerted a negative regulatory effect on the protein levels of VEGFA (Figure 4C,D), indicating that miR-221 targets VEGFA to negatively regulate its expression.

## Discussion

Herein, we successfully isolated and identified EPCs from the peripheral blood of patients with CSF. Atorvastatin EPC treatment induced increases in VEGFA protein levels, cell proliferation, and cell migration. miR-221 expression was down-regulated by atorvastatin treatment; miR-221 overexpression in EPCs exerted opposing effects to those of atorvastatin treatment on VEGFA protein levels, cell proliferation, and cell migration. The protective EPC effect of atorvastatin treatment on the VEGFA protein expression could be significantly suppressed by miR-221 overexpression. miR-221 directly bound the VEGFA 3′UTR to inhibit its expression.

As we have mentioned, EPCs are essential for maintaining normal endothelial function [11,28]. Hill *et al* measured flow-mediated brachial arterial reactivity and found a close association between the number of circulating EPCs and the combined Framingham risk factor score and endothelial function among patients with different cardiovascular risk degrees but no history of cardiovascular events [29]. Moreover, Werner et al confirmed that the circulating EPC level predicts the incidence of cardiovascular diseases and death caused by cardiovascular events and that it can be used to identify the patients with increased risk of cardiovascular diseases [13]. According to the report of Minami et al the numbers of EPCs in patients with coronary heart disease were up-regulated by atorvastatin lipid-lowering therapy [24]. In addition, Oikonomou et al reported that a large dose of atorvastatin could up-regulate the circulating EPC number in patients with ischemic heart failure, which is highly beneficial to maintain a normal endothelium function [30]. In agreement with those findings, we observed a significant inducible effect of atorvastatin treatment on EPC proliferation and migration, indicating that atorvastatin may improve CSF by inducing EPC proliferation and migration. More importantly, atorvastatin treatment also significantly increased the VEGFA protein level in EPCs, suggesting the involvement of VEGFA in the atorvastatin effects on EPC proliferation and migration.

Many studies have focused on the role of miRNAs in the biological modulation of mammalian blood vessels. miRNAs could modulate cell proliferation, migration, apoptosis, and capillary formation, thereby regulating endothelial angiogenesis [31,32]. A total of 15 miRNAs, including miR-221 and miR-222 have been found to be highly expressed within human umbilical vein endothelial cells (HUVECs) [23]. By far, the most typical miRNAs include miR-221, miR-222, miR-130a, miR-126 and miR-92a [23,33–36]. By investigating and comparing the expression levels of miR-221/222 within tissues and within circulation in patients with and without significant atherosclerosis, Bildirici et al revealed that miR-221 serves as an underlying prognostic biomarker of the progression of local atherosclerosis [27]. However, the angiogenesis effect of miR-221 in cardiovascular disease is controversial. Some studies had found that miR-221 has an inhibitory effect on angiogenesis in cardiovascular disease. Poliseno et al. reported that miR-221/miR-222 negatively regulate HUVECs vessel formation by targeting c-Kit [23], And Minami et al discovered that miR-221/222 expression levels were significantly higher in patients with CAD and that they were negatively correlated with EPC numbers [24]. Zhang et al found significantly higher miR-221 expression levels in patients with atherosclerosis than in controls, and they found that miR-221 overexpression dramatically decreased EPCs proliferation [26]. The above results are consistent with ours. However, some studies have obtained the opposite results Ni et al showed that miR-221 overexpression using a mimic promoted the angiogenic capacity of EPCs [37]. And, cervical cancer cell-secreted exosomal miR-221 promoted angiogenesis of microvascular endothelial cells in cervical cancer [38]. We have observed alterations in the expression of various key genes participating in embryonic angiogenesis, such as VEGF, Flt1 (VEGF-R1), flk1 (VEGF-R2), and Tie1 [39]. After discussing the evidence for atorvastatin treatment increasing these VEGFA protein levels in EPCs, we will discuss our finding on how miR-221 could affect EPC proliferation and migration via VEGFA. In contrast to its effects on VEGFA, miR-221 expression was significantly down-regulated by atorvastatin treatment; opposing to atorvastatin treatment, miR-221 overexpression in EPCs inhibited VEGFA protein levels, cell proliferation, and cell migration. More importantly, the overexpression of miR-221 could significantly suppress the atorvastatin-induced increases in VEGFA protein levels, as well as the growth and migratory capacity of EPCs, indicating that miR-221 regulation of VEGFA mediates atorvastatin effects on EPCs.

Regarding molecular mechanisms, miRNAs could bind the 3′UTRs of downstream protein-coding mRNAs to exert negative regulatory effects on their stability and translation, thus modulating cell growth, cell differentiation, apoptosis and other biological processes [40,41]. Herein, we predicted an miR-221 binding site on the VEGFA mRNA 3′UTR using online tools. Then, we used a luciferase reporter assay to show that miR-221 could negatively regulate the expression of VEGFA by targeting its 3′UTR. In conclusion, atorvastatin improves the cell proliferation and migration of EPCs via an miR-221/VEGFA axis. Therefore, atorvastatin may be considered a potent agent against CSF pending further *in vivo* and clinical investigation.

## Abbreviations

CAD: coronary artery disease
CAM: chick allantoic membrane
CSF: coronary slow flow
EPC: endothelial progenitor cell
HRP: horseradish peroxidase
HUVEC: human umbilical vein endothelial cell
IF: immunofluorescence
miRNA: microRNA
MTT: 3-(4,5-Dimethylthiazol-2-yl)-2,5-diphenyltetrazolium bromide
VEGFA: vascular endothelial growth factor-A
